# Spatial analysis of colorectal cancer incidence and proportion of late-stage in Massachusetts residents: 1995–1998

**DOI:** 10.1186/1476-072X-6-20

**Published:** 2007-06-04

**Authors:** Laurie M DeChello, T Joseph Sheehan

**Affiliations:** 1 Department of Community Medicine and Health Care, University of Connecticut School of Medicine, 263 Farmington Ave, Farmington, CT 06030-6325, USA

## Abstract

**Background:**

The aims of this study were to determine if observed geographic variations in colorectal cancer incidence are simply random or are statistically significant deviations from randomness, whether statistically significant excesses are temporary or persistent, and whether they can be explained by risk factors such as socioeconomic status (SES) or the percent of the population residing in an urban area rather than a rural area. Between 1995 and 1998, 6360 male and 6628 female invasive colorectal cancer cases were diagnosed in Massachusetts residents. Cases were aggregated to Census tracts and analyzed for deviations from random occurrence with respect to both location and time.

**Results:**

Six geographic areas that deviated significantly from randomness were uncovered in the age-adjusted analyses of males: three with higher incidence rates than expected and eight lower than expected. In the age-adjusted analyses of females, one area with a higher incidence rate, and one area with a lower incidence rate than expected, were found. After adjustment for SES and percent urban, some of these areas were no longer significantly different.

**Conclusion:**

Public health practitioners can use the results of this study to focus their attention onto areas in Massachusetts that need to increase colorectal screening or have elevated risk of colorectal cancer incidence.

## Background

This observational epidemiological study of Massachusetts colorectal cancer incidence and proportion of late-stage examines the geographic variations over a four-year period. The investigation looks to determine whether observed excesses of incidence rates or proportion of late-stage cases occur at random or represent statistically significant deviations from randomness using purely spatial and space-time models. By modeling space and time, it is possible to determine whether these excesses are stable over time, or only temporary. The models were also adjusted for SES and percent urban to see if these factors could account for the significantly high and low areas. The study is part of the surveillance process in which the data are analyzed to identify areas where closer attention is needed and to aid in determining the need for public health programs or evaluate ones that are in place.

A spatial analysis of a portion of Cape Cod, Massachusetts studied 1983–1986 and found no statistically significant variation of colorectal cancer incidence [1]. There have not been any studies published and indexed in PubMed that include all of Massachusetts in a spatial scan analysis evaluating colorectal cancer incidence. However, studies have looked into how factors such as socioeconomic status (SES) and urban/rural classifications of Census tracts help to explain why an area is high in colorectal incidence. Although Williams, et al.[2] found there to be no urban-rural gradient or social class associations to colorectal cancer incidence in Scotland, other studies have found increased incidence of colon cancer to be associated with urban areas [3,4]. A literature review assessing association of SES and cancer risk found a fairly consistent increased risk of colon cancer with increased SES [5]. Pollack, et al. reported higher proportions of late stage diagnoses of colorectal cancer in low income patients in California [6]. Although Rushton, et al. did not find clear urban/rural patters in Iowa, they did report that high proportion of late-stage diagnoses where patients traveled longest distances between where they lived and were diagnosed [7].

The current study examines the colorectal cancer incidence and proportion of late-stage colorectal cancer of Massachusetts residents diagnosed between 1995 and 1998. Males and females were analyzed separately using Poisson regression and the spatial scan statistic. This was performed to give a more complete picture of spatial and spatial-temporal occurrence than was previously available and to provide this information to the Massachusetts Cancer Registry to aid in cancer control efforts.

## Results

### Poisson regression

The Poisson regression of male invasive colorectal cancer and the covariates, wealth, poverty and percent urban, found that although there wasn't a decreasing or increasing trend of incidence by SES category, the estimates for categories 1 through 4 for both wealth and poverty were higher than for category 5. Therefore, both SES components were dichotomized so that categories 1 through 4 equal a new category 1, and category 5 equals a new category 2. Table 1 displays the percent of increased risk of colorectal cancer for males living in tracts with category 1 for both SES components compared to males living in tracts with a category 2. Neither SES component was a statistically significant predictor of male colorectal cancer incidence in the Poisson regression. However, these dichotomized SES components were included in the SaTScan models when they were adjusted for SES and for SES with percent urban. Percent urban had a parameter estimate of -0.906 and was statistically significant (p-value < 0.0001).

**Table 1:** Relative change in male and female colorectal incidence.

Variable	Categories Compared	Percent Increase
Males		
Wealth	1–2	16.7%
Poverty	1–2	10.1%
Females		
Wealth	1–5	1.9%
Wealth	2–5	4.0%
Wealth	3–5	0.0%
Wealth	4–5	-3.5%
Poverty	1–2	34.2%

The assessment of two levels of wealth and poverty for males and five levels of wealth and 2 levels of poverty for females where category 1 of wealth and the higher category of poverty are the highest levels of wealth and poverty. For example, for males, tracts with a wealth category of 1 had higher wealth compared to category 2 and 16.7% more colorectal incidence.

The Poisson regression of female invasive colorectal cancer and the covariates showed that wealth did not follow a trend (see Table 1), nor were categories 1 through 4 all higher than category 5. Category 4 was actually lower risk than category 5. Therefore, the 5 categories were not dichotomized and were all included when wealth was included as a covariate in the SaTScan models adjusted for SES and for SES with percent urban. Wealth was not a significant predictor of female colorectal cancer in the Poisson regression. The poverty component of SES parameter estimates of categories 1 through 4 indicated higher colorectal risk than category 5. Therefore, it was dichotomized where categories 1 through 4 were collapsed. Poverty was a statistically significant predictor of female colorectal cancer (p-value < 0.0001). Both SES components were entered as covariates in the spatial scan models. Percent urban had a parameter estimate of 0.522 with a p-value of < 0.0001, which can be interpreted as meaning the more urban the tract, the higher the colorectal incidence.

### Purely spatial analyses of male colorectal cancer incidence

**Figure 1:**
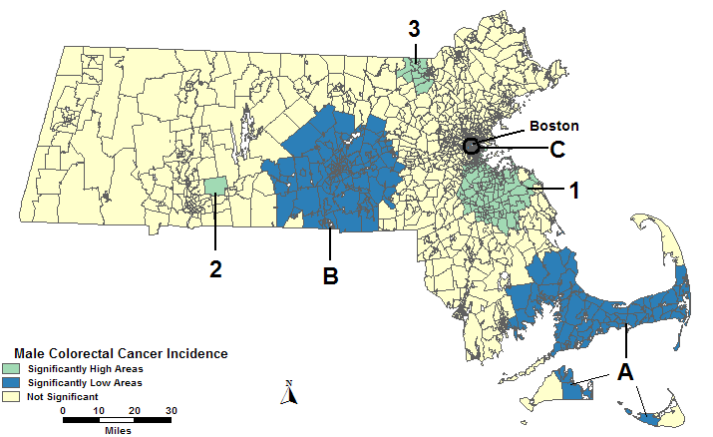
**Purely spatial, males, no covariate adjustment**. Purely spatial analysis of male colorectal cancer incidence without covariate adjustment, 1995–1998.

In the purely spatial analysis of males without covariates, 3 high areas and 3 low areas were found to be statistically significantly different than the rest of the state. High 1 in Figure 1, southeast of Boston, had 783 cases when only 615 were expected. High 2 is a small geographic area in western Massachusetts with 3.24 times more cases than expected. High 3, along the New Hampshire border, had 57% more cases than expected. The most statistically significant low, Low A, covers most of Cape Cod and portions of Nantucket and Martha's Vineyard; it had 30% fewer cases than expected. Low B, centered on Worcester, and Low C, in and around Boston, also had fewer cases than expected. The statistics for all purely spatial analyses can be found in Table 2.

**Table 2:** Male purely spatial analysis.

	High Areas			Low Areas		
	1	2	3	A	B	C
No Covariates						
Observed	783	39	127	331	489	76
Expected	615.0	12.0	81.0	476.5	609.2	129.5
RR	1.27	3.24	1.57	0.70	0.803	0.59
p-value	< 0.0001	< 0.0001	0.0318	< 0.0001	0.0027	0.0051
Urbanicity						
Observed	767	39	80	339	215	76
Expected	619.8	16.2	45.5	458.9	293.2	131.3
RR	1.24	2.40	1.76	0.74	0.73	0.58
p-value	< 0.0001	0.0261	0.0466	< 0.0001	0.0172	0.0028
SES						
Observed	783	39	423	331	350	*
Expected	626.6	12.6	336.3	494.4	465.1	*
RR	1.25	3.11	1.26	0.67	0.75	*
p-value	< 0.0001	< 0.0001	0.0438	< 0.0001	< 0.0001	*
SES & Urbanicity						
Observed	755	*	*	339	489	*
Expected	629.8	*	*	468.2	594.1	*
RR	1.20	*	*	0.72	0.82	*
p-value	0.0061	*	*	< 0.0001	0.0437	*

Male colorectal cancer incidence statistics for the purely spatial analyses, Massachusetts, 1995–1998.

*Area not significant for this analysis.

When percent urban was added as a covariate to the purely spatial analysis of males, High 1 changed shape slightly and the relative risk (RR) was reduced slightly. High 3 shifted to the southwest and had an increased RR. A tract was added to Low A and increased the RR. Low B greatly reduced in geographic size and shifted its center east of Worcester; its RR decreased. High 2 and Low C remained the same geographically; however, High 2 had a reduced RR.

When the SES components were added as covariates, Highs 1 and 2 remained the same geographically with only small reductions in the RRs compared to the analysis without covariates. High 3 greatly increased in geographic size with a reduced RR. Low A was identical to that in Figure 1 with a slightly reduced RR. Low B shifted to the southwest with a RR lower by 0.05. Low C was not statistically significant in the analysis adjusting for SES.

**Figure 2:**
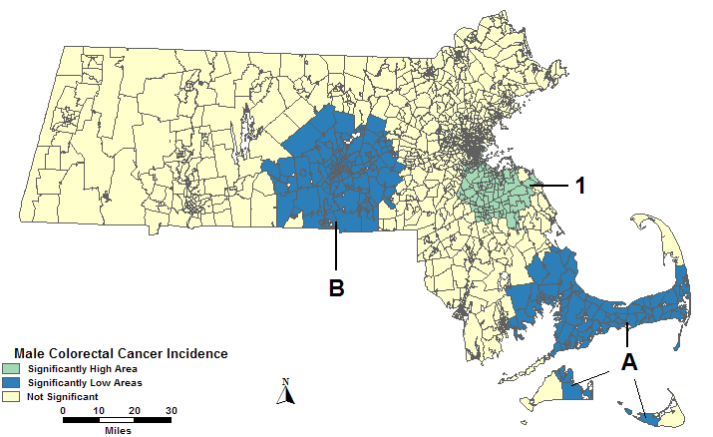
**Purely spatial, males, multiple adjustments**. Purely spatial analysis results of male colorectal cancer incidence adjusted for socio-economic status and percent urban, 1995–1998.

Figure 2 displays results from the analysis of males adjusted for percent urban and SES together, which found High 1 to change shape with a slightly reduced RR. Low A was identical geographically to the area found in the percent urban alone adjusted analysis. Low B was identical to the analysis without covariates. High 2, High 3, and Low C were not statistically significant in the analysis adjusted for percent urban and SES.

### Space-time analyses of male colorectal cancer incidence

The space-time analysis of males without covariates found all areas but High 3 to be identical to the purely spatial analysis displayed in Figure 1 since the entire study period was found to be more statistically significant than any part of the time frame for those areas. Only 1996 to 1998 were found to be statistically significant in the space-time analysis for High 3; the geographic area is the same as in Figure 1, but with a lower RR, 1.38. These and the statistics for all space-time analyses can be found in Table 3.

**Table 3:** Male space-time analyses. Male colorectal cancer incidence statistics for the space-time analyses, Massachusetts, 1995–1998.

	High			Low		
	1	2	3	A	B	C
Time Frame	95–98	95–98	96–98	95–98	95–98	95–98
No Covariates						
Observed	783	39	348	331	489	76
Expected	615.0	12.0	252.6	476.5	609.2	129.5
Relative Risk	1.27	3.24	1.38	0.70	0.80	0.59
p-value	< 0.0001	0.0003	0.0016	< 0.0001	0.0205	0.0409
Urbanicity						
Observed	767	*	110	199†	*	*
Expected	619.8	*	62.7	298.2†	*	*
Relative Risk	1.24	*	1.75	0.667†	*	*
p-value	0.0002	*	0.0098	< 0.0001†	*	*
SES						
Observed	783	39	348	331	350	*
Expected	626.6	12.6	252.3	494.4	465.1	*
Relative Risk	1.25	3.11	1.38	0.67	0.75	*
p-value	< 0.0001	0.0007	0.0011	< 0.0001	0.0015	*
SES & Urbanicity						
Observed	755	*	*	199†	*	*
Expected	629.8	*	*	304.7†	*	*
Relative Risk	1.20	*	*	0.65†	*	*
p-value	0.0438	*	*	< 0.0001†	*	*

*Area not significant for this analysis.

† The time frame for this area in these particular analyses was 96–98

The space-time analysis of males adjusted for percent urban found High 1 to change shape, with only a slight reduction in the RR from the space-time analysis without covariates. High 3 greatly increased in size geographically with a larger RR. Low A has a different statistically significant time frame in the percent urban adjusted analysis, 1996–1998; its RR is only slightly reduced. High 2, Low B, and Low C were not statistically significant in this analysis.

**Figure 3:**
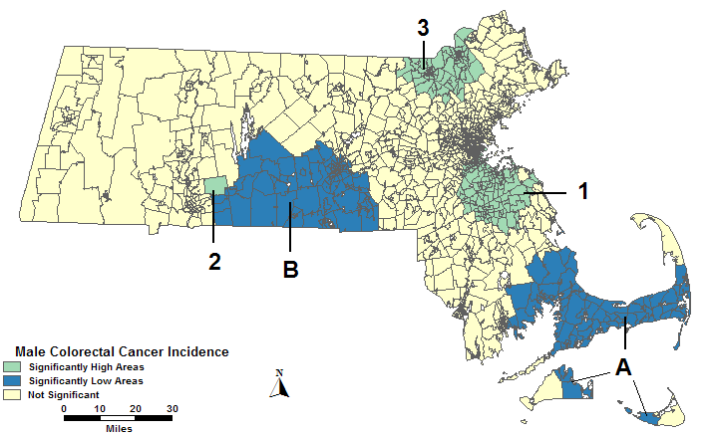
**Space-time, males, adjusted**. Space-time analysis results of male colorectal cancer incidence adjusted for socio-economic status, 1995–1998.

Figure 3 displays the SES adjusted space-time analysis, which found High 1, High 2, High 3, and Low A to be identical to the space-time analysis without covariates with slightly reduced or identical RRs. Low B shifted to the southwest with a slightly lower RR. Low C was not statistically significant in the SES adjusted analysis.

In the space-time analysis of males adjusted for both percent urban and SES, High 1 had a different shape than the analysis without covariates, and a slightly lower RR. Low A was statistically significant for the same time frame as in the analysis adjusted for percent urban alone: 1996–1998. High 2, High 3, Low B, and Low C were not statistically significant in the percent urban and SES adjusted analysis.

### Purely spatial analyses of female colorectal cancer incidence

**Figure 4:**
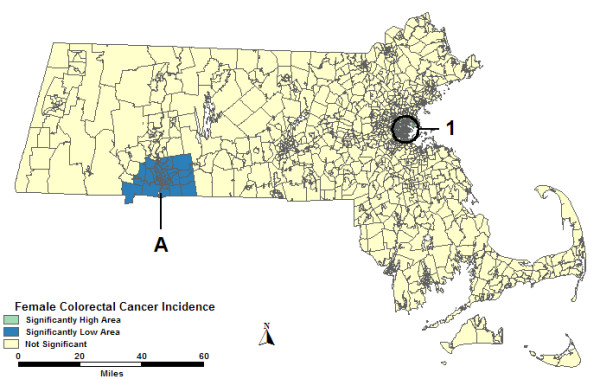
**Purely spatial, females, no covariate adjustment**. Purely spatial analysis of female colorectal cancer incidence without covariate adjustment, 1995–1998.

The purely spatial analysis of females without covariates found 1 high and 1 low area statistically significantly different from the rest of the state. High 1 in Figure 4 was in and around Boston with 57% more cases than expected. Low A in southwestern Massachusetts had 24% fewer cases than expected. The statistics for all purely spatial analyses of females can be found in Table 4.

**Table 4:** Female colorectal cancer incidence statistics for the purely spatial analyses, Massachusetts, 1995–1998

	High 1	Low A
No Covariates		
Observed	442	403
Expected	281.2	533.0
Relative Risk	1.57	0.76
p-value	< 0.0001	< 0.0001
Urbanicity		
Observed	442	403
Expected	291.3	507.1
Relative Risk	1.52	0.80
p-value	< 0.0001	0.0103
SES		
Observed	442	403
Expected	281.7	574.5
Relative Risk	1.57	0.74
p-value	< 0.0001	< 0.0001
SES & Urbanicity		
Observed	442	333
Expected	292.1	430.1
Relative Risk	1.51	0.77
p-value	< .0001	0.0079

Both the purely spatial analysis of females adjusted for percent urban and the analysis adjusted for SES found the identical two areas in Figure 4 to be statistically significant. The RRs are similar or the same as in the analysis without covariates. The purely spatial analysis of females adjusted for percent urban and SES together found the identical High 1 as in Figure 4. However, Low A reduced in size geographically, which did not significantly change the RR.

### Space-time analyses of female colorectal cancer incidence

The entire study period was statistically significant in the space-time analysis without covariates for both High 1 and Low A, as seen in Figure 4. Therefore, the statistics of these two areas are identical to the purely spatial analysis without covariates, which are displayed for all space-time analyses of females in Table 5.

**Table 5:** Female colorectal cancer incidence statistics for the space-time analyses, Massachusetts, 1995–1998.

	High 1	Low A
Time Frame	95–98	95–98
No Covariates		
Observed	442	403
Expected	281.2	533.0
Relative Risk	1.57	0.76
p-value	< 0.0001	0.0002
Urbanicity		
Observed	442	*
Expected	291.3	*
Relative Risk	1.52	*
p-value	< 0.0001	*
SES		
Observed	442	403
Expected	281.7	547.5
Relative Risk	1.57	0.74
p-value	< 0.0001	< 0.0001
SES & Urbanicity		
Observed	442	*
Expected	292.1	*
Relative Risk	1.51	*
p-value	< 0.0001	*

*Area not significant for this analysis

The percent urban adjusted space-time analysis of females found High 1 to cover the same tracts as the purely spatial analysis without covariates. The RR was slightly reduced. Low A was not statistically significant in the analysis adjusted for percent urban. The SES adjusted space-time analysis found both High 1 and Low A to be the same geographically as the analysis without covariates, as displayed in Figure 4. The analysis adjusted for percent urban and SES found High 1 to be the same as the analysis adjusted for percent urban. However, Low A was not statistically significant for the analysis adjusted for both percent urban and SES.

### Proportion of late-stage analyses

The purely spatial and space-time analyses of the proportion of late-stage colorectal cancer was not adjusted for age due to small numbers of late-stage cases by age group within tracts. Both the purely spatial and space-time of proportion of late-stage analyses resulted in no areas of excess being statistically significant for males and females. The purely spatial analysis of males found only one area to be lower than expected, but the p-value was 0.9994, not even close to being significant. The space-time analysis of males and both analyses of females did not even find any areas with a rank less than 9999.

## Discussion

There are a couple possibilities as to why the proportion of late-stage analyses did not find any statistically significant areas. There may not have been enough power to detect statistical significance, or late-stage cases may not be geographically clustered. The results support the latter theory since the purely spatial analysis of males only found one non-significant cluster with a p-value just below 1, and the other analyses did not find any non-significant clusters.

This study uses the number of diagnoses of colorectal cancer as a proxy for incidence in the state of Massachusetts. It is possible that areas in this study reflecting average or low rates of colorectal cancer may truly be higher if cases are not being detected. Therefore, these areas should be investigated to determine if there could be protective factors so that cases are not occurring or if in reality there are cases going undiagnosed. Areas exhibiting high rates of cancer may be due to colorectal cancer screening promotions in these communities. Consequently, the results need to be interpreted with caution.

The current study adjusted for age, SES, and percent urban. Other known risk factors could be used in such a study and possibly explain the high areas uncovered. The following attributable risk percentages for such factors have been reported in the literature: 9% due to HRAS1 alleles[8], 6% to 33% due to low levels of physical activity [9-11], 39% due to low intake of beta-carotene, 14% due to low intake of vitamin C, 4% due to high intake of seasoning fats in Italy [12], 4% to 17% due to high frequency of red meat consumption [9,12], 2% to 14% due to low consumption of fruits and vegetables [9,13], 3% to 39% in males and 1% to 11% in females due to alcohol intake [9,14], 6.7% due to a body mass index = 25 [15], and 4% to 11% due to family history[12,14,16]. Strong associations were also found between smoking and rectal cancer [17-19], as well as GSTM1 and GSTT1 polymorphisms with colon cancer[20].

### Limitations

Addresses were contracted out by MCR to companies that geocode them and appended Census tracts based on those geocoded locations. Addresses that are geocoded into the wrong tract could potentially create areas of statistically significant excess that are not truly excesses. This is especially problematic with smaller populations or with addresses where more cases are likely to come from, such as a long-term care facility.

Post office box addresses were not geocoded to a Census tract. These cases were either put into the one tract covering the town containing that post office or, where the post office's town contained multiple tracts, were randomly assigned to Census tracts within that town. The town that contained the patient's post office box may not be the town in which the patient actually lived. Most post office addresses occur in large cities. However, since cities have more cases compared to medium or smaller towns, a few post office address cases are not going to determine if a cluster is statistically significant or not. Data was not provided regarding the ungeocoded cases as to if the difficulty in geocoding was an address that did not exist or a post office box. The relative risks of the statistically significantly elevated areas of the unadjusted analyses were calculated while omitting those cases originally ungeocoded. The RRs for the male high areas would be reduced by either 0.08 or 0.07, while the female high area would be reduced by 0.18.

The use of both 1990 and 2000 Census population data assume a gradual change in population over the decade. However, it is important to note that an abrupt change can occur in the population distribution when facilities close (e.g., a military base or long-term care facility) or are opened (retirement villages or a correctional facility). Such was the case for High 2 in the male analyses. The analysis at the tract-level is sensitive to these abrupt changes, which may have made High 2 artificially elevated.

## Conclusion and recommendations

High 1 in the females and High 1 in the males do not change after adjustment for SES and percent urban. Perhaps a case-control study could be designed to determine what other risk factors might be elevating the incidence rate in these areas.

High 2 in the males and Low A in the females were no longer significant due to adjustment with percent rural. Low A from the females covers the geographic area of High 2 in the males. It is interesting that the same area could have conflicting rates based on gender. Perhaps there is a cultural issue at play where the males are much more likely than females to be screened for colorectal cancer in this area. Low B in the males also is no longer significant when adjusted for percent urban, but only in the space-time analysis.

High 3 and Low C in males are not significant after adjustment for SES and percent urban. Low A in males remains unchanged in the purely spatial analysis but reduces in time frame after adjusting for SES and percent urban. These areas should be investigated to determine what it is about the interaction with SES and percent urban that affects these areas.

This study was intended to facilitate aiming the focus of public health practitioners towards areas that need their attention. Not only are the high areas of colorectal cancer incidence in need of investigation, but the low areas as well since there might be cases evading detection. Some of the differences in colorectal incidence rates might be due to unequal access to diagnostic equipment and screening programs. Like analyses adjusting for other known risk factors, such as those listed above, would be very useful in investigating the remaining areas of excess and low incidence rates to help determine why they vary from what is expected. This is a useful tool for analyzing surveillance data.

## Methods

The data are from the Massachusetts Cancer Registry (MCR): 6360 male and 6628 female invasive incident colorectal cancer cases of known stage diagnosed between 1995 and 1998. The case record was designed to include information on place of residence at the time of diagnosis classified according to the minor civil division (town), ZIP Code, and Census tract, as well as the age at diagnosis, date of diagnosis, race, and stage of colorectal cancer where stage was the historical Surveillance, Epidemiology and End Results (SEER) summary stage: local, regional, distant and unknown. Cases staged as unknown were not included in these analyses. Regional and distant stages were considered late-stage for the proportion of late-stage analyses. Of the 12,988 cases, 12,222 were in white patients.

### Aggregation unit

Census tracts were used to geographically aggregate the data. Since 9.6% of the cases diagnosed in 1995–1998 were not assigned a reliable Census tract by MCR due to the absence of an address, an incorrect address, or a post office box mailing address, these cases needed to be assigned a Census tract. Town and Census tract boundaries were compared to assign the unassigned cases to tracts. For a town containing two or more Census tracts, the cases were randomly assigned to tracts within the town based on the proportion of the town's population each tract contributed. There were 456 male and 512 female cases that needed to be randomly assigned, or 7.2% and 7.7% of all male and female cases, respectively. More detail on how assignment of cases missing tract was performed has been previously published [21].

### Spatial analyses

Population data are from the 1990 [22] and 2000 [23] Decennial censuses. All SaTScan analyses were performed using age-adjusted expected case counts in place of the population counts. To calculate the age-adjusted expected counts, the 1990 and 2000 male and female population counts were combined into a weighted average of the two based on the years being analyzed: 1995 through 1998. This was done for each age group within each tract. The natural log of this weighted average was entered as the offset variable in a Poisson regression in SAS [24]. There were a few tracts with a zero population for a certain age group; a population of one was entered for these so a log could be taken. The Poisson regression included age as an independent variable and number of cases within each tract and age group as the dependent variable to calculate the age-adjusted expected counts. The expected counts were aggregated across age groups within each tract and multiplied by 1000 for the population file for all spatial analyses.

The SaTScan software [25] was used to perform the spatial analyses and assumes that incident colorectal cancer follows a Poisson distribution. According to the null hypothesis, the probability of a case being diagnosed in a particular location is equal throughout the state, based primarily on the density of the population.

In all analyses, the number of Monte Carlo replications was set to 9,999. Space-time analyses were performed so that the regional variations over the entire time period, 1995–1998, could be analyzed in a single model. The space-time analyses utilized the entire study period's information (1995–1998) but looked for clusters that were significant for smaller time frames up to and including the entire study period. Purely spatial analyses were also performed, which do not take time into account. The maximum spatial cluster size was first set to include up to 25% of the population to detect both excesses and deficits together, and then set at 10% to test for excesses and deficits separately. Testing at the 10% level identified smaller, more defined areas. However, to adjust for multiple testing, each area had a likelihood associated with it that was compared to the 9,999 likelihoods from the initial 25% maximum spatial size test. The maximum temporal cluster size was set at 90% and also included purely spatial clusters with temporal size of 100% for all space-time analyses.

The overall socioeconomic status (SES) and percent urban status of each tract were determined and included as covariates separately and together along with age to determine if SES or percent urban could account for the high or low areas. An SES index was created using the method of Yost and colleagues in a principal component analysis using varimax rotation (the variables used in several methods were explored to determine which group of variables accounted for more variance. Since the Yost method accounted for more variance, it was utilized in this study) [26]. Two components accounted for about 80% of the variance among the seven economic measures obtained from the Census. The first component explained 49.1% of the variance and was made up of median income, median rent, median house value, and percent with at least a high school diploma from the 1990 Census [22], and will be referred to as wealth. The second component explained 31.0% of the variance made up of the percent unemployed, percent working class, and percent below the poverty level, and will be referred to as poverty. The two scores from the principal component analysis were included in the SaTScan analyses as covariates in SES adjusted analyses along with percent urban.

Percent urban was created by using data from the 2000 Census. The Census Bureau provides an urban and rural population breakdown for each tract. The percent urban was calculated by dividing the population of the urban area of the tract by the sum of the population of both the urban and rural areas of the tract. This percent urban classification was included in SaTScan analyses as a covariate by itself and along with the SES components.

Poisson regression was performed using the SES scores and percent urban as predictors of the number of incident cases within tracts for males and females, separately, but all age groups combined. This analysis was performed using PROC GENMOD in SAS [24]. SES scores were categorized into approximately equal sized quintiles.

## Authors' contributions

TJS: PI, responsible for design, funding, of project with overall responsibility for implementing the project, including the final paper. LMD: Principal data analysis, responsible for final checks on accuracy of data and all analyses, including their written interpretation. Both authors have read and approved the final manuscript.
